# The relationship between hope, medical expenditure and survival among advanced cancer patients

**DOI:** 10.3389/fpsyg.2023.1151976

**Published:** 2023-05-23

**Authors:** Junxing Chay, Vinh Anh Huynh, Yin Bun Cheung, Ravindran Kanesvaran, Lai Heng Lee, Chetna Malhotra, Eric Andrew Finkelstein

**Affiliations:** ^1^Lien Centre for Palliative Care, Duke-NUS Medical School, Singapore, Singapore; ^2^Program in Health Services and Systems Research, Duke-NUS Medical School, Singapore, Singapore; ^3^Centre for Quantitative Medicine, Duke-NUS Medical School, Singapore, Singapore; ^4^Division of Medical Oncology, National Cancer Centre, Singapore, Singapore; ^5^Department of Haematology, Singapore General Hospital, Singapore, Singapore

**Keywords:** cancer, hope, survival, healthcare utilization, healthcare expenditure

## Abstract

**Introduction:**

Among those with advanced illness, higher levels of hope may offer physiological benefits. Yet, greater levels of hope may also encourage aggressive treatments. Therefore, higher levels of hope may lead to greater healthcare utilization, higher expenditure, and longer survival. We test these hypotheses among patients with advanced cancer.

**Methods:**

A secondary data analysis from a cross-sectional survey of 195 advanced cancer patients with high mortality risk linked to subsequent healthcare utilization (outpatient, day surgeries, non-emergency admissions), health expenditures, and death records. The survey collected data on hope, measured generally by the Herth Hope Index (HHI) and more narrowly by two questions on illness-related hope. Generalized linear regression and Cox models were used to test our hypotheses.

**Results:**

142 (78%) survey participants died during the period of analysis, with close to half (46%) doing so within a year of the survey. Contrary to expectation, HHI scores did not have a significant association with healthcare utilization, expenditure or survival. Yet, illness-related hope, defined as those who expected to live at least 2 years, as opposed to the likely prognosis of 1 year or less as determined by the primary treating oncologist, had 6.6 more planned hospital encounters (95% CI 0.90 to 12.30) in the 12-months following the survey and 41% lower mortality risk (hazard ratio: 0.59, 95% CI 0.36 to 0.99) compared to those who were less optimistic. Secondary analysis among decedents showed that patients who believed that the primary intent of their treatment is curative, had higher total expenditure (S$30,712; 95% CI S$3,143 to S$58,282) in the last 12 months of life than those who did not have this belief.

**Conclusion:**

We find no evidence of a relationship between a general measure of hope and healthcare utilization, expenditure, or survival among advanced cancer patients. However, greater illness-related hope is positively associated with these outcomes.

## 1. Introduction

Hope is a valuable coping strategy among those with life limiting illnesses. Hope has been shown to mediate the relationship between physical health and psychological distress, and improve life satisfaction, spiritual well-being, and health related quality of life (Korner, 1970; Chi, 2007; Rustøen et al., 2010). Hope has also been posited to provide psychoneuroimmunological benefits that may lead to better health outcomes, including greater survival among those with advanced illnesses (Price et al., 2016; Corn et al., 2022). For example, Antoni et al. argue that feelings of optimism can reduce chronic stress and that this can lead to improved sympathetic nervous system signaling, reduced hypothalamic pituitary adrenal axis dysregulation and inflammation, and increased cellular immunity and that these factors could inhibit tumor growth among cancer patients (Antoni et al., 2006, 2009).

Although hope has many benefits, it may also increase the likelihood of engaging in motivated reasoning and self-deception, both of which increase the likelihood of several cognitive biases (Kunda, 1990; Martindale, 2005). One such bias is optimism bias, which makes people believe that they are less likely to experience an adverse outcome than evidence would support (McNeil et al., 1982; Weinstein, 1987; Jansen et al., 2016). A related bias is termed the illusion of superiority. In this case people understand the risks, but believe that their own outcome will be far better than that of the average patient (Buunk and Van Yperen, 1991). Patients may also suffer from misattribution bias, where they confound their current health state with their long-term prognosis, even if the two are unrelated (Nisbett and Wilson, 1977). This misattribution may be increasingly common with the growing availability of treatments that effectively palliate symptoms but only marginally improve prognoses (Davis et al., 2017).

The relationship between hope and several cognitive biases was formally tested among 200 advanced cancer patients with physician-estimated prognoses of 1 year or less in a recent cross-sectional study entitled Survival expectations and Hope Among Cancer Patients at End-of-Life (SHAPE) (Finkelstein et al., 2021). The authors showed that higher levels of hope, as measured by the Herth Hope Index (HHI) (Herth, 1992), was associated with greater levels of self-deception, optimism bias, illusion of superiority, and misattribution (Finkelstein et al., 2021). Others have also shown a direct correlation between hope and positive perceptions of prognosis (Seyedrasooli et al., 2014) and between hope and optimism (Alarcon et al., 2013). Although undoubtedly mediated by health communication between patients and providers, higher levels of hope may partly explain why patients with advanced cancer worldwide tend to overestimate their prognoses (Weeks et al., 1998; Granek et al., 2013; Chen et al., 2016).

These findings suggest that, among those with advanced cancer, more hopeful patients may be more likely to pursue aggressive and expensive treatments (Weeks et al., 1998) which could lead to better outcomes (Temel et al., 2010; Cardona-Morrell et al., 2016). Yet, the evidence on these relationships is mixed. For example, Price et al. found that higher levels of optimism was associated with longer survival (HR = 0.80; CI 0.65–0.97), although they did not explore whether this was mediated by health seeking behavior (Price et al., 2016). Yet, Temel et al. (2010) showed that more aggressive treatments did not lead to better health outcomes. They showed that advanced cancer patients receiving palliative care had longer survival than those receiving life extending treatment. Although others have come to different conclusions (Calvo-Espinos et al., 2015; Xu et al., 2018).

To date, no studies have simultaneously explored the relationship between hope, health seeking behavior, and survival. By linking the SHAPE survey data with 4 years of follow-up data that includes healthcare utilization, health expenditures, and death records, we test (1) whether more hopeful patients have greater planned healthcare utilization and expenditure and (2) whether they have a survival advantage. Results provide valuable information on the relationship between hope and survival among patients with advanced care, and the mediating effect of health seeking behaviors.

## 2. Methods

### 2.1. Study sample

This study extends the analyses conducted in the prior SHAPE study. As described in Finkelstein et al. (2021) SHAPE is a cross-sectional survey administered to 200 adult patients with advanced cancers (either advanced Stage IV solid cancer or an advanced stage of leukemia or lymphoma cancer) who were Singapore citizens or permanent residents, and with a likely prognosis of 1 year or less as determined by the primary treating oncologist. Other inclusion criteria included (a) age ≥ 21 years old, (b) diagnosed with advanced/stage IV solid cancer, leukemia or lymphoma, (c) mentally competent and aware of their condition. Patients were recruited between June 2018 and May 2019 at outpatient oncology clinics and inpatient wards at the Singapore General Hospital (SGH) and National Cancer Centre Singapore (NCCS). Initially, 293 patients were referred by oncologists, of whom 200 consented and completed the survey. Informed consent was obtained and the survey was administered by trained interviewers in either English or Mandarin, depending on the patient's preference, using tablets. In 2021, the study team reconsented 51 patients of the 69 patients still alive to have their medical and billing records up to March 2022 linked to survey responses (13 rejected, and 5 were uncontactable). Records of the 131 patients who died in 2021 or earlier were also linked. In total, 182 patients were included in the healthcare utilization/expenditure analysis and 195 patients were included in the survival analysis. By March 2022, when the study was completed, 142 of the 195 patients had died. The study was approved by the SingHealth Institutional Review Board (CIRB Ref. No: 2017/2181).

### 2.2. Healthcare utilization/expenditure and survival

Healthcare utilization and expenditure were obtained from billing records of visits to specialist outpatient clinics, day surgery procedures, Accident & Emergency (A&E) visits, inpatient admissions, and pharmacy prescriptions. As public hospitals in Singapore are not expected to turn a profit, we used gross non-subsidized prices to proxy for the true cost to the health system. Our analysis focused on planned utilization received as more hopeful patients are expected to have greater planned utilization but may or may not have greater unplanned utilization. Planned utilization is defined as visits to outpatient oncology clinics, day surgeries and inpatient admissions not through the A&E department. A&E visits and admissions through the A&E are assumed to be unplanned. Information on patient's dates of death was extracted from medical records. Expenditures were reported in Singapore dollars (S$).

We calculated healthcare utilization and expenditures for the first 12 months after survey completion for those who survived at least 12 months after the survey or the last 12 months of life for those who died within 12 months after being surveyed (henceforth “12-month period around the survey”). In an additional analysis, we restricted the sample to the deceased only and calculated healthcare utilization and expenditures during their last 12 months of life. The former allows us to analyze the full sample but may not capture all aggressive treatments. The latter focuses on the period when more hopeful patients are most likely to pursue aggressive but marginally effective treatments.

### 2.3. Measures of hope

Hope was assessed using the abbreviated version of the Herth Hope Index (HHI) (Herth, 1992). The Herth Hope Index (HHI) is an adapted from the Herth Hope Scale (HHS) and designed to be used in clinical settings. The instrument includes 12 items, grouped into three subgroups to measure inner sense of temporality and future, inner positive readiness and expectancy and interconnectedness with self and others (Herth, 1992). The items were graded on a 4-point scale labeled: Strongly disagree, Disagree, Agree, Strongly agree. Scores range from 12 to 48 with higher scores indicating higher degree of hopefulness. For the Mandarin speakers, we used a validated version obtained from a published psychometric evaluation of the translated instrument (Chan et al., 2012). As hope is a composite construct that may comprise many dimensions, we also examined two measures that are more closely associated with their medical condition/treatment (henceforth referred to as “illness-related hope”). These include expecting to live at least 2 years and believing that the primary intent of treatment is to be cancer free (Finkelstein et al., 2021). Patients' expectation of survival duration was assessed based on patients' answers to the question “Do you expect to be alive in …?” with options including “20 years”, “10 years”, “5 years”, “2 years”, “1 year” “9 months” and “6 months”. Treatment intent was based on the question “What do you think is the primary goal of your treatment regimen?” with response options of “Cure my illness so I will be cancer free at some point”, “Prolong my life”, “Manage my symptoms (e.g., control pain)”, “Don't know” and “Others”.

### 2.4. Clinical factors

The survey also asked patients to report time since first diagnosis, primary cancer site (gastrointestinal, genitourinary, leukemia/lymphoma, lung, prostate and others), stage at first diagnosed (only for solid cancers), and self-reported health rating on the day of the interview. Socioeconomic data such as age, gender, education level and marital status were also included. The full instrument is included in Supplementary material Full Survey Instrument.

### 2.5. Statistical analysis

We separately estimated the effect of hope using regressions models that control for age, gender, educational attainment [primary school or below ( ≤ 6 years), secondary school (≤ 10 years), and post-secondary school (>10 years)], self-reported health rating of the day, cancer site, whether cancer was detected at an early stage, and years since cancer diagnosis. Robust standard errors were reported for all regressions.

Utilization, measured in terms of the number of visits or admissions, was estimated using a negative binomial regression. For expenditure, we used a generalized linear model (GLM) with log link and gamma-distributed errors. For each outcome of interest, we calculated the average marginal effects of hope as the average difference in predicted outcomes for a marginal change in HHI, a higher HHI quartile relative to the 1st HHI quartile (1st quartile: 15–35, 2nd quartile: 36–42, 3rd quartile: 43–46, 4th quartile: 47–48) to capture potential non-linearities. A similar approach was used to calculate the average marginal effects of illness-related hope (i.e. expecting to be alive in at least 2 more years and believing the primary intent of treatment is to become cancer-free).

To investigate for a potential survival advantage, we first examined Kaplan-Meier (KM) survival curves stratified by (1) HHI quartile subgroups and (2) presence of illness-related hope, and tested for differences in survival using the log-rank test. Adjusting for the same set of potential confounders as the healthcare utilization/expenditure analysis, we then fit a Cox proportional-hazards regression model, with separate hazard functions stratified by leukemia and non-leukemia cancers. This was because leukemia status failed the Schoenfeld residuals test of proportionality at the 5% significance level.

Our base case survival analysis conservatively assumes that self-rated health is a confounder influencing both hope and survival. However, one may argue for exclusion because self-rated health may also be an intermediate variable between hope and survival such that controlling for self-rated health absorbs the effects attributable to hope. Therefore, in additional analyses, we omitted self-rated health from the set of controls as a robustness check.

## 3. Results

### 3.1. Descriptive analysis

Table 1 describes the characteristics of our study sample. The final analytical sample included 195 participants for the survival analysis and 182 participants for the utilization/expenditure analysis. The mean age was 65.8 (SD: 65.7) and the majority were Chinese (80%), male (68%, SD: 46%), married (82%, SD: 38%) and diagnosed with solid cancer (90%). Close to two-thirds were at late stage of cancer (Stage IV) (62%) and close to half were initially diagnosed at an advanced stage (45%). The top three cancer sites were genitourinary (23%), lung (21%) and prostate (21%). By 2022, 142 (78%) participants had passed away, with close to half (46%) doing so within a year of the survey.

**Table 1:** Summary of patient characteristics and outcomes (*n* = 195).

**Variables**	**Mean (SD)/*n* (%)**
**Patient characteristics**	
Age	65.7 (10.5)
Female	61 (31%)
Ethnicity	
Chinese	155 (79%)
Malay	19 (10%)
Others	21 (11%)
Married	160 (82%)
Education level	
Primary or below	66 (34%)
Secondary	67 (34%)
Post-secondary	62 (32%)
Patient is diagnosed with:	
Solid cancer	173 (89%)
Liquid	22 (11%)
Stage of initial cancer diagnosis	
Early stages (I-III)	43 (22%)
Advanced stage (IV)	85 (44%)
Liquid cancer	22 (11%)
Don't know	45 (23%)
Cancer site	
Gastrointestinal	28 (14%)
Genitourinary	44 (23%)
Leukemia/Lymphoma	26 (13%)
Lung	41 (21%)
Others	12 (6%)
Prostate	44 (23%)
Health rating today: 0 (worst imaginable)−10 (best imaginable)	6.3 (2.2)
**Hope measures**	
Herth Hope Index	39.7 (7.5)
Herth hope index by quartile:	
1st quartile (12–35)	51 (26%)
2nd quartile (36–42)	58 (30%)
3rd quartile (43–46)	45 (23%)
4th quartile (47–48)	41 (21%)
Expect to be alive in at least 2 more years	180 (92%)
Believe primary intent of treatment is to become cancer-free	80 (41%)
**Healthcare utilization and expenditures 1 year from survey**	
**(*****n** =* **182)**	
Number of planned healthcare visits	28.9 (15.6)
Planned healthcare expenditure, S$	83,940 (94,808)
Total healthcare expenditure, S$	101,816 (109,663)
**Healthcare utilization and expenditures in the last year**	
**of life (*****n** =* **142)**	
Number of planned healthcare visits	29.1 (16.6)
Planned healthcare expenditure, S$	78,126 (85,078)
Total healthcare expenditure, S$	104,730 (109,066)
**Survival outcomes**	
Deceased participants	142 (78%)
Survival duration since time of survey	
< 1 year	84 (46%)
1–2 years	31 (17%)
>2 years	67 (37%)

Within the 12-month period following the survey, patients incurred an average medical expenditure of S$101,816 (SD: S$109,663). On average, patients had 28.9 (SD: 15.6) planned visits/admissions costing S$83,940 (SD: S$94,808). In the last 12 months of life, decedents (*n* = 142) incurred an average medical expenditure of S$104,730 (SD: S$109,066). These patients averaged 29.1 (SD: 16.6) planned visits/admissions costing S$78,126 (SD: S$85,078).

**Figure 1:**
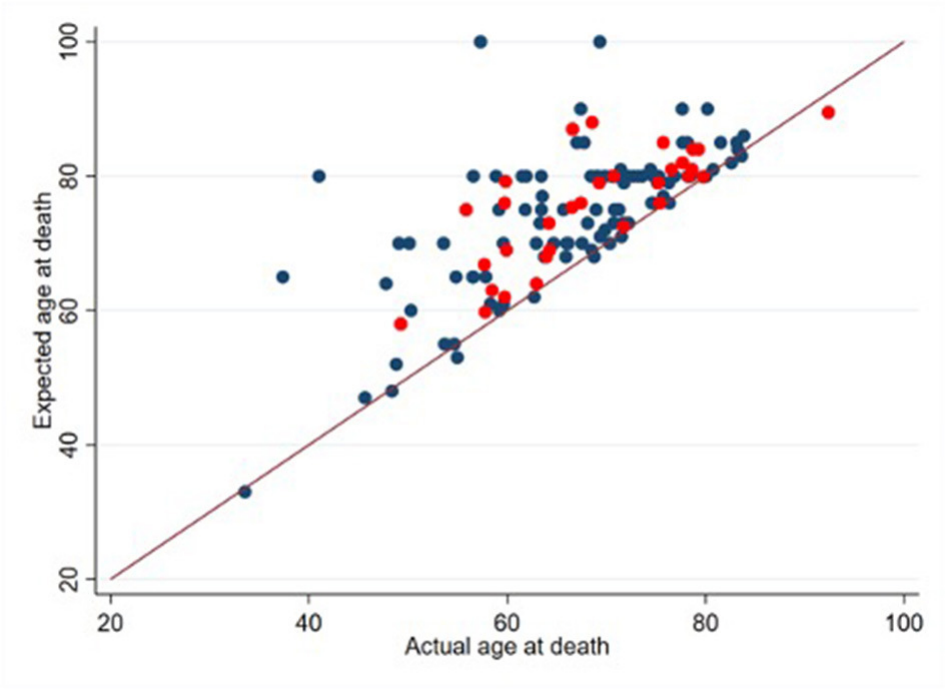
Expected vs. actual age at death (*n* = 132). Patients who provided an estimate of their age at death are denoted with blue points (*n* = 101). Patients who provided an estimate of their minimum survival period are denoted with red points (*n* = 31). 10 decedents did not provide an estimate of their age at death or their minimum survival period.

The mean HHI score was 39.5 (SD: 7.5) out of 48. Cronbach's alpha for the study sample was 0.88 for both the English and Chinese versions. Nearly all patients (180, 92%) stated that they expected to live at least 2 more years and 80 (42%) participants believed that the primary goal of treatment was to become cancer-free. In reality, about 50% of participants passed away within 2 years of taking the survey. Figure 1 provides a visual representation of the overestimation of survival outcomes.

### 3.2. Effects on healthcare utilization and expenditure

Table 2 reports the average marginal effects of hope on annual healthcare utilization and expenditure within the 12-months of taking the survey. Contrary to expectations, we did not find that HHI had a statistically significant association on the frequency of planned care or healthcare expenditure. The average marginal effect of HHI was small and not statistically significant. Quartile-specific estimates suggested that a non-linear relationship may be driving this null finding, but these estimates were generally not significantly different from the first quartile at the 5% level. Although difficult to explain, patients in the 3rd quartile of HHI (HHI 43–46) had the lowest total healthcare expenditure among the quartiles, S$29,157 lower on average compared to the 1st HHI quartile (95% confidence interval (CI) –S$60,255 to S$1,941, *p*-value = 0.07).

**Table 2:** Effect of hope on healthcare utilization, expenditure and survival.

**Dependent variable**	**Number of planned visits/admissions^a^**	**Planned visits/admissions expenditure^b^**	**Total expenditure^b^**	**Survival^c^**
	Mean, (95% CI)	Mean, S$ (95% CI)	Mean, S$ (95% CI)	Hazard ratio, (95% CI)
HHI	0.07 (−0.28 to 0.43)	−59 (−1,421 to 1,302)	−537 (−2,018 to 943)	0.992 (0.968 to 1.016)
HHI quartile (ref. 1st quartile)				
2nd quartile	0.46 (−5.34 to 6.25)	7,471 (−22,614 to 37,556)	3,182 (−29,420 to 35,784)	1.096 (0.682 to 1.762)
3rd quartile	−1.89 (−8.94 to 5.15)	−14,171 (−47,979 to 7,283)	−29,157^*^ (−60,255 to 1,941)	0.783 (0.470 to 1.306)
4th quartile	1.23 (−6.14 to 8.61)	−20,348 (−33,725 to 23,575)	−8,901 (−41,748 to 23,946)	1.298 (0.787 to 2.142)
Expect to be alive in at least 2 more years	6.60^**^ (0.90 to 12.30)	11,112 (−19,026 to 41,249)	3,320 (−34,390 to 41,030)	0.592^**^ (0.355 to 0.987)
Believe primary intent of treatment is to become cancer-free	2.76 (−1.57 to 7.10)	3,069 (−17,383 to 23,522)	11,147 (−12,016 to 34,310)	1.268 (0.898 to 1.792)
Number of observations	179	179	179	193

Healthcare utilization and expenditures were measured in 12-month period around the survey. All models are controlled for age, gender, education, cancer site, whether cancer was detected at early stage, years since cancer diagnosis, and health rating today. CI, Confidence interval. Statistical significance denoted by ^*^p < 0.1, ^**^p < 0.05.

a Average marginal effects from negative binomial regression model.

b Average marginal effects from generalized linear model (GLM) with log link and gamma-distributed errors.

c Hazard ratios on all-cause mortality using Cox proportional-hazards regression model stratified by leukemia/lymphoma cancer site.

Patients who expected to live at least 2 more years had 6.6 more planned visits/admissions (23% increase; 95% CI 0.90 to 12.30, *p*-value = 0.02) on average compared to those who did not. Believing that the primary intent of treatment is to become cancer-free did not have a significant effect on planned visits/admissions. Neither measure of illness-related hope had a statistically significant association with either planned or total healthcare expenditure. However, point estimates suggested that patients who expected to live at least 2 more years had on average *higher* planned and total medical expenditure (S$11,112 and S$3,320 more respectively) than those who expected to live fewer than 2 years. Those who believe that the primary intent of treatment is to become cancer-free also had *higher* planned and total medical expenditure (S$3,069 and S$11,147 more respectively) than those who did not have this belief.

In efforts to provide further insight into the results, we restricted the analysis to the decedents-only sample and in the last 12 months of life. The results are generally similar with those based on the full sample (see Supplementary Table A1). HHI did not have a statistically significant association with frequency of healthcare utilization and expenditure, although point estimates for HHI quartiles showed a more consistent trend of *fewer* planned visits/admissions and expenditure among more hopeful patients, which is contrary to our initial hypothesis. Neither measure of illness-related hope was statistically associated with planned healthcare utilization or expenditure. Nevertheless, point estimates remained positive implying that more optimistic patients were more likely to utilize planned care, which is consistent with expectations. Patients who believed that the primary intent of treatment was to become cancer-free spent significantly more in total medical expenditure (S$30,712, 95% CI S$3,143 to S$58,282, *p*-value = 0.03) compared to those who did not believe.

### 3.3. Effects on survival

**Figure 2:**
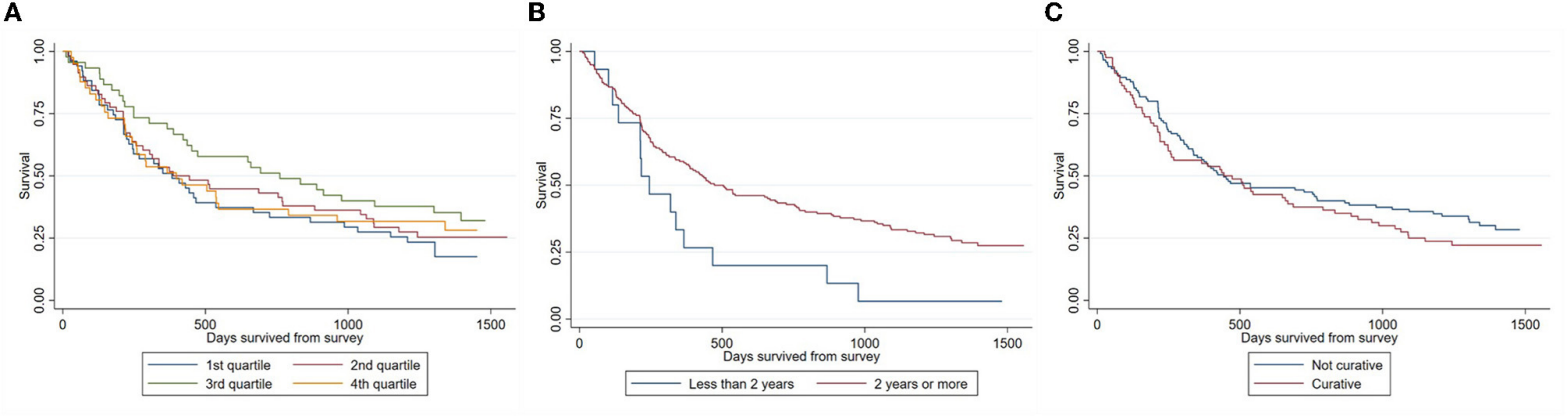
Stratified Kaplan-Meier survival estimates. **(A)** By HHI quartiles; **(B)** by survival expectations; **(C)** by belief of treatment's primary intent.

Figure 2 presents the KM survival curve of patients, stratified by measures of hope and optimism. Consistent with our initial hypothesis, survival is trending upward for the first three quartiles. However, contrary to expectations, it then drops for the fourth quartile. Regardless, survival functions were not statistically different across HHI quartiles (*p-*value = 0.38), as determined by the log-rank test. The survival of those who expected to live at least 2 more years was higher than those who did not (*p-*value = 0.02), but not those who believed that the primary intent of treatment is to become cancer-free (*p-*value = 0.59).

Adjusting for confounders in the stratified Cox regression did not change our results (Table 2). Only higher survival expectations had a significant association with actual survival. Those who expected to live at least 2 more years had a 40% lower hazard of death than those who did not (hazard ratio (HR): 0.592, 95% CI 0.355 to 0.987, *p-*value = 0.04). Excluding self-rated health as a covariate in the Cox regression model showed that one point increase in HHI was associated with 2.2% points lower hazard of death (HR: 0.978, 95% CI 0.956 to 0.999, *p-*value = 0.03) and that the 3rd HHI quartile had 36.0% lower hazard of death as compared to the 1st HHI quartile HR: 0.640, 95% CI 0.396 to 1.035, *p-*value = 0.08) (Supplementary Table A2). There was no change to the estimated effects of biases (Supplementary Table A2).

## 4. Discussion

### 4.1. Main results

This study investigated the relationship between hope, health seeking behavior and survival among advanced cancer patients. Contrary to expectations, we did not find a clear positive relationship between a broad measure of hope and these outcomes. We found that the 4th HHI quartile (those with HHI scores of the maximum possible or one point below) incurred *lower* planned and total healthcare expenditure and also had *lower* survival odds compared to the other quartiles, although the differences were not statistically significant and may simply be due to chance. These results are consistent with prior studies. Petticrew et al. examined 26 studies focusing on two aspects of hope, including fighting spirit and helplessness/hopelessness and found no significant associations with survival (Petticrew et al., 2002). We did find that excluding self-rated health as a covariate showed that higher hope was significantly associated with greater survival, but our study design did not allow us to discern if self-rated health is an intermediate variable that should be excluded, or a confounder that should be included.

The lack of associations may result because hope, as measured by the HHI, may be more distal compared to our measures of illness-related hope. We found some evidence that illness-related hope was associated with greater healthcare utilization/spending and a greater survival advantage. Patients who expected to live for at least 2 more years had more frequent planned healthcare utilization in the 12-month period around the survey, although this did not translate into significantly higher healthcare expenditure. Furthermore, patients who believed that the primary intent of treatment is to become cancer-free spent S$30,712 more on total healthcare in their last 12 months of life than those who did not.

Although our study did not capture communication between providers and patients, other studies reveal that providers are often hesitant to convey negative information to patients so as not to dampen hope (Weeks et al., 1998). Our results suggest these concerns may be well founded. We found that patients who expected to live for at least 2 more years had greater survival than those who did not. This finding is consistent with Lee et al. who also found that optimistic expectations were associated with better survival (Lee et al., 2003). Yet, this survival increase was not without cost as we also showed that an unintended consequence of this potentially unfounded optimism was to increase healthcare utilization and expenditures at end-of-life.

### 4.2. Study limitations

This analysis has several limitations that need to be considered in the context of these results. First, we focused on measures of hope at a single point in time. Yet Solano et al. found that hope changes with illness (Solano et al., 2016). A review by Kylma et al. further showed that hope is related to temporal factors such as pain and quality of life (Kylm et al., 2009). Prognostic beliefs have also been shown to change over time (Ozdemir et al., 2023) and may be influenced by both health status and prior treatment decisions (Finkelstein et al., 2021). For example, Echarte et al. proposed that self-deception entails a lower cognitive load than accepting the truth and argues that, as a result, patients can become more hopeful as illness progresses (Echarte et al., 2016). Relatedly, our hypothesis assumes that patients' primary hope is for a cure, or at least for significant life extension. This is likely true upon diagnosis and consistent with our prior findings showing the relationship between hope and several biases. Nevertheless as illness progresses, hope may shift toward more obtainable goals, such as finalizing affairs and being cared for at a place of choice (Duggleby and Wright, 2004; Clayton et al., 2005). Similarly, availability, accessibility and quality of end-of-life care, including conversations about palliative care, may shift patients' hope and intention away from aggressive care and life extension (Earle et al., 2008).

Second, we were also unable to rule out reverse causality between hope and healthcare utilization/spending. This could result, for example, if frequent hospitalizations induce the illusion of treatment success which then influences decisions for further treatment. Conversely, hope may decrease after rounds of unsuccessful treatments, which could lead to decisions to suspend efforts to extend life.

Third, healthcare utilization/expenditure and survival duration may be censored for patients who stopped receiving treatment at SGH or NCCS because they transferred to a private care setting. Although we were told by the clinicians that these cases are rare, our estimates could potentially be biased if more hopeful patients are systematically more likely to transfer out of SGH or NCCS. However, we suspect this is unlikely to be an issue as NCCS cares for 70–80% of cancer patients in Singapore and we did not find any significant association between HHI and the likelihood of a dying outside SingHealth network of care providers.

Finally, our measures of hope are imperfect. HHI, which is one of several available hope indices (Bryant and Harrison, 2015), is a broad measure of hope encompassing three dimensions: temporality and future, positive readiness and expectancy, and interconnectedness (Herth, 1992). It is possible that results would differ if an alternative measure of hope were employed. On the other hand, hope indicators more closely related to participants' condition could be the result of biases or misinformation, potentially arising from poor communication with providers (Weeks et al., 1998). We were unable to capture what information was conveyed by the providers to the patients. Due to litigation risk resulting from nondisclosure (Austin, 2019), it is likely that patients were informed about prognosis and treatment intent. However, the extent of disclosure and whether or not patients truly understood what was conveyed is unclear.

## 5. Conclusion

This is one of the first studies to investigate the relationship between hope, health seeking behavior, and survival. We found evidence suggesting that the presence of illness-related hope among advanced cancer patients is associated with better survival, but also greater planned healthcare utilization, and health expenditures. Future work should explore how to improve patient-provider communication with the goal of sustaining hope in patients, while mitigating biases that may influence treatment decisions.

## Data availability statement

De-identified data supporting the conclusions of this article will be made available to researchers with an approved IRB in place.

## Ethics statement

The studies involving human participants were reviewed and approved by SingHealth Institutional Review Board (CIRB Ref. No: 2017/2181). The patients/participants provided their written informed consent to participate in this study.

## Author contributions

EF and JC contributed to conception and design of the study. LL and RK organized the database and collected the data. VH, JC, and EF performed the statistical analysis and wrote the first draft of the manuscript. LL, RK, YC, and CM critically revised the manuscript. All authors contributed to manuscript revision, read, and approved the submitted version.
