# Supplementary material for: The relationship between hope, medical expenditure and survival among advanced cancer patients
Source: Front Psychol. 2023 May 23;14:1151976. doi: 10.3389/fpsyg.2023.1151976 (PMC10242009; doi:10.3389/fpsyg.2023.1151976)
Supplement: Supplementary file 1 [file Data_Sheet_1.pdf]

## APPENDIX

**Table A1. Effect of hope on healthcare utilization and expenditure in the last 12 months of life (n = 138)**

| Dependent variable | Number of planned visits/ admissions[a] Mean, (95% CI) | Planned visits/ admissions expenditure[b] Mean, S$ (95% CI) | Total expenditure[b] Mean, S$ (95% CI) |
|---|---|---|---|
| HHI | -0.09 (-0.45 to 0.27) | -695 (-2,048 to 657) | -773 (-2,429 to 883) |
| HHI quartile (ref. 1st quartile) | | | |
| 2nd quartile | -3.35 (-9.52 to 2.81) | -6,976 (-36,009 to 22,057) | -11,490 (-43,778 to 20,798) |
| 3rd quartile | -3.89 (-12.14 to 4.36) | -18,854 (-50,047 to 12,340) | -26,072 (-63,702 to 11,558) |
| 4th quartile | -1.67 (-10.00 to 6.65) | -16,532 (-44,849 to 11,786) | -17,875 (-53,690 to 17,940) |
| Expect to be alive in at least 2 more years | 3.66 (-3.58 to 10.89) | 9,296 (-25,142 to 43,734) | 4,388 (-44,643 to 53,418) |
| Believe primary intent of treatment curative | 3.27 (-2.31 to 8.84) | 18,931 (-4,935 to 42,796) | 30,712** (3,143 to 58,282) |

Notes: Healthcare utilization and expenditures were measured one year from the survey. All models are controlled for age, gender, education, cancer site, whether cancer was detected at early stage, years since cancer diagnosis, and health rating today. CI: Confidence interval. Statistical significance denoted by * p<0.1, ** p<0.05, *** p<0.01.

[a] Average marginal effects from negative binomial regression model.

[b] Average marginal effects from generalized linear model (GLM) with log link and gamma-distributed errors.

**Table A2. Effect of hope on survival**

| **Dependent variable** | **Survival[a]** Hazard ratio (95% CI) |
|---|---|
| HHI | 0.978** (0.956 to 0.999) |
| HHI quartile (ref. 1st quartile) | |
| 2nd quartile | 1.026 (0.639 to 1.650) |
| 3rd quartile | 0.640* (0.396 to 1.035) |
| 4th quartile | 0.964 (0.570 to 1.630) |
| Expect to be alive in at least 2 more years | 0.511*** (0.309 to 0.846) |
| Believe primary intent of treatment is to become cancer-free | 1.342* (0.948 to 1.899) |
| Number of observations | 195 |

Notes: All models are controlled for age, gender, education, cancer site, whether cancer was detected at early stage, and years since cancer diagnosis. CI: Confidence interval. Statistical significance denoted by * $p<0.1$, ** $p<0.05$, *** $p<0.01$.

[a] Hazard ratios on all-cause mortality using Cox proportional-hazards regression model stratified by leukemia/lymphoma cancer site.

# Survival Expectations and Hope Among Cancer Patients at End-of-Life (SHAPE)

*Set B: Self Beliefs, Incentive Compatible*

**For interviewer: Fill in the following information after you have completed the questionnaire.**

PARTICIPANT CODE: (FROM CONSENT FORM) [ ] DATE OF INTERVIEW (DD/MM/YYYY): _____/_____/_____

TIME STARTED: _______ TIME ENDED: ________ TOTAL INTERVIEW TIME: _____________ MIN

INTERVIEWER'S NAME: ______________________________

NAME OF HOSPITAL:

☐1 NATIONAL CANCER CENTRE

☐2 SINGAPORE GENERAL HOSPITAL

PATIENT'S GENDER: ☐1 MALE ☐2 FEMALE

PATIENT'S DATE OF BIRTH (MM/YYYY): _____/_____

SURVEY CONDUCTED IN : ☐1 ENGLISH

☐2 MANDARIN (DIALECT __________________)

**Q1. Are you currently enrolled in any clinical trial?**

*(Interviewer: Clinical trials involve the testing of **new treatments** on some patients before these treatments are made available to other patients in general. Do not include observational studies for this).*

1. Yes
2. No
3. I don't know

| INTRODUCTION |
|---|
| We are conducting a survey with patients being treated at this hospital to assess their beliefs about their own health and illness. **We recognize that many questions are very sensitive. Please skip those that you do not feel comfortable answering**. Your responses and those of others like you will help to us develop strategies in the future that better inform patients with similar illnesses. Your identity and the information given will be kept strictly confidential. Only group data will be reported. |

| **SECTION A: BASIC INFORMATION** | | |
|---|---|---|
| **A1.** | Have you ever been diagnosed with cancer? (either solid or liquid) | |
| | ☐1 | Yes |
| | ☐2 | No |
| | ☐3 | Don't know |

**[For the interviewer: If A1≠1, please terminate the survey]**
**[If patient is diagnosed with solid cancer, continue to ask A2 to A4, otherwise, skip to A4]**

| | | |
|---|---|---|
| **A2.** | Do you know the current stage of your cancer? | |
| | ☐1 | Early Stage (Stage I, II or III) |
| | ☐2 | Advanced Stage (Stage IV/metastatic) |
| | ☐3 | Don't know |
| | ☐4 | Other, please specify _______________________ |
| **A3.** | What was the stage of your cancer when you **first** learned about it? | |
| | ☐1 | Stage I |
| | ☐2 | Stage II |
| | ☐3 | Stage III |
| | ☐4 | Stage IV/metastatic |
| | ☐5 | Don't know |
| | ☐6 | Other, please specify _______________________ |
| **A4.** | In which year were you first diagnosed with cancer? ☐ | |

## SECTION B: FUNCTIONAL STATUS, QUALITY OF LIFE & MENTAL WELL-BEING

| **Your Health and Well-Being** |
|---|
| This survey asks for your views about your health. This information will help keep track of how you feel and how well you are able to do your usual activities. *Thank you for completing this survey!*<br><br>For each of the following questions, please mark an ☒ in one box that best describes your answer. |

**B1.** In general, would you say your health is:

| Excellent | Very good | Good | Fair | Poor |
|---|---|---|---|---|
| ☐1 | ☐2 | ☐3 | ☐4 | ☐5 |

**B2.** The following questions are about activities you might do during a typical day. Does <u>your health now limit you</u> in these activities? If so, how much

| | Yes, limited a lot | Yes, limited a little | No, not limited at all |
|---|---|---|---|
| a. <u>Moderate activities</u> such as moving a table, pushing a vacuum cleaner, bowling, or do tai chi. | ☐1 | ☐2 | ☐3 |
| b. Climbing <u>several</u> flights of stairs. | ☐1 | ☐2 | ☐3 |

B3. During the <u>past 4 weeks</u>, have you had any of the following problems with your work or other regular daily activities <u>as a result of your physical health</u>?

| | All of the time | Most of the time | Some of the time | A little of the time | None of the time |
|---|---|---|---|---|---|
| a. <u>Accomplished less</u> than you would like | ☐1 | ☐2 | ☐3 | ☐4 | ☐5 |
| b. Were limited in the <u>kind</u> of work or other activities | ☐1 | ☐2 | ☐3 | ☐4 | ☐5 |

**B4.** During the <u>past 4 weeks</u>, have you had any of the following problems with your work or other regular daily activities <u>as a result of any emotional problems</u> (such as feeling depressed or anxious)?

| | All of the time | Most of the time | Some of the time | A little of the time | None of the time |
|---|---|---|---|---|---|
| a. <u>Accomplished less than</u> you would like | ☐1 | ☐2 | ☐3 | ☐4 | ☐5 |
| b. Did work or activities <u>less carefully than usual</u> | ☐1 | ☐2 | ☐3 | ☐4 | ☐5 |

**B5.** During the <u>past 4 weeks</u>, how much did <u>pain</u> interfere with your normal work (including work outside the home and housework)?

| Not at all | A little bit | Moderately | Quite a bit | Extremely |
|---|---|---|---|---|
| ☐1 | ☐2 | ☐3 | ☐4 | ☐5 |

| **B6.** The questions are about how you feel and how things have been with you <u>during the past 4 weeks</u>. For each question, please indicate one number that comes closest to the way you have been feeling. | | | | | |
|---|---|---|---|---|---|
| | All of the time | Most of the time | Some of the time | A little of the time | None of the time |
| a. Have you felt calm and peaceful? | ☐1 | ☐2 | ☐4 | ☐5 | ☐6 |
| b. Did you have a lot of energy? | ☐1 | ☐2 | ☐4 | ☐5 | ☐6 |
| c. Have you felt down-hearted and blue? | ☐1 | ☐2 | ☐4 | ☐5 | ☐6 |

| **B7.** During the <u>past 4 weeks</u>, how much of the time has your <u>physical health or emotional problems</u> interfered with your social activities (such as visiting friends, relatives, etc.)? | | | | |
|---|---|---|---|---|
| All of the time | Most of the time | Some of the time | A little of the time | None of the time |
| ☐1 | ☐2 | ☐3 | ☐4 | ☐5 |

*Thank you for completing these questions!*

SF-12v2® Health Survey © 1995, 2004 Medical Outcomes Trust and QualityMetric Incorporated. All rights reserved.

SF-12® is a registered trademark of Medical Outcomes Trust.

(SF-12v2® Health Survey Standard, Singapore (English))

| | |
|---|---|
| **B8.** | Please rate your pain by circling the one number that tells how much pain you have **right now.**<br><br>0 (**No pain**) 1 2 3 4 5 6 7 8 9 10 (**Pain as bad as you can imagine**) |
| **B9.** | We would like to know how good or bad your **health is TODAY**. This scale is numbered from 0 to 10. 10 means the best health you can imagine and 0 means the worst health you can imagine. Indicate on the scale how your **health is TODAY**.<br><br>0 (**Worst imaginable health**) 1 2 3 4 5 6 7 8 9 10 (**Best imaginable health**) |

The next three items **(B10 to B12)** are about **how you feel about different aspects of your life.** Please indicate one number per line to describe how often you feel that way.

| | | **Hardly ever** | **Some of the time** | **Often** |
|---|---|---|---|---|
| **B10.** | How often do you feel that you lack companionship? | 1 | 2 | 3 |
| **B11.** | How often do you feel left out? | 1 | 2 | 3 |
| **B12.** | How often do you feel isolated from others? | 1 | 2 | 3 |

**SECTION C: INTENT OF TREATMENT**

| | | |
|---|---|---|
| **C1.** | What do you think is the **primary** goal of your treatment regimen? | |
| | ☐1 | Cure my illness so I will be cancer free at some point |
| | ☐2 | Prolong my life |
| | ☐3 | Manage my symptoms (e.g., control pain) |
| | ☐4 | Don’t know |
| | ☐5 | Other, please specify ______________________________ |

**C2.** Do you believe that your current treatment regimen will…?

| | | **Yes** | **No** | **Don’t know** |
|---|---|---|---|---|
| 1. | Prolong your life | 1 | 2 | 3 |
| 2. | Manage your symptoms | 1 | 2 | 3 |

**C3.** *Because we are most interested in your answer to the following question, if your prediction matches that of your treating clinician (which we will find out at a later date), we will donate $25 to charity.*

Please indicate which charity organization you would like to donate money to.
☐1 Singapore Cancer Society
☐2 HCA Hospice Care
☐3 Red Cross

Do you believe that your current treatment regimen will…?

| | **Yes** | **No** | **Don’t Know** |
|---|---|---|---|
| Cure your illness | 1 | 2 | 3 |

**SECTION D: PREDICTIONS FOR SURVIVAL**

Some of the oldest people in the world have lived well into their 100's. But nobody lives forever. In fact, few people expect to live past 100. In the next few questions, we will ask your beliefs regarding your own survival.

We recognize that these are difficult questions and appreciate your willingness to take part in this survey. Remember you can skip any questions you do not feel comfortable answering.

**D1.** Did you know that prior research shows that most patients are overly optimistic about how long they are likely to live?

| | |
|---|---|
| ☐1 | Yes, I was aware of that |
| ☐2 | No, I had not heard that |

*Yes, data reveal that patients tend to over-estimate the effectiveness of cancer treatments. Please consider this fact and answer based on what you believe to be your most likely outcome.*

**D2.** Using the scale below: Indicate on the line your **current age.**

20 30 40 50 60 70 80 90 100

**D2.1** *Because we are most interested in your answer to the following question, if your prediction matches that of your treating clinician (which we will find out at a later date), we will donate another $25 to charity.*

Please indicate which charity organization you would like to donate money to.

☐1 Singapore Cancer Society
☐2 HCA Hospice Care
☐3 Red Cross

Indicate on the line how old you think you might be **when you die**.

20 30 40 50 60 70 80 90 100

**D3.** Many factors may influence survival outcomes. Some are known, such as diet, exercise, and stress levels, but many remain unknown.

Compared to other patients being treated with the **same condition as you**, do you expect your survival outcomes to be…?

| | |
|---|---|
| ☐1 | Much longer than the average |
| ☐2 | Slightly longer than the average |
| ☐3 | About the same |
| ☐4 | Slightly less than the average |
| ☐5 | Much less than the average |
| ☐6 | Don't know |

**D4.** Do you expect to be alive in …?

| | | | | |
|---|---|---|---|---|
| D4.1 | 20 years | | | |
| | ☐1 | Yes (Skip to D5) | ☐2 | No |
| D4.2 | 10 years | | | |
| | ☐1 | Yes (Skip to D5) | ☐2 | No |

| | | | | | |
|---|---|---|---|---|---|
| | D4.3 | 5 years | | | |
| | | ☐1 | Yes (Skip to D5) | ☐2 | No |
| | D4.4 | 2 years | | | |
| | | ☐1 | Yes (Skip to D7) | ☐2 | No |
| | D4.5 | 1 year | | | |
| | | ☐1 | Yes (Skip to D7) | ☐2 | No |
| | D4.6 | 9 months | | | |
| | | ☐1 | Yes (Skip to D7) | ☐2 | No |
| | D4.7 | 6 months | | | |
| | | ☐1 | Yes (Skip to D7) | ☐2 | No (Skip to D7) |

| | | |
|---|---|---|
| **D5.** | Please check the box that you believe most likely reflects your **cancer status FIVE years** from now. | |
| | ☐1 | I will be cancer free (cured, remission, undetectable in the body) |
| | ☐2 | I will not be cancer free but my cancer will be controlled with treatment. |
| | ☐3 | My cancer will have spread but I am likely to be alive. |
| | ☐4 | I am unlikely to be alive. |
| **D6.** | We would like to know how good or bad your **health** will be in **FIVE years**. This scale is numbered from 0 to 10. 10 means the best health you can imagine and 0 means the worst health you can imagine while still being alive. Indicate on the scale how your **health** will be in **FIVE years**. | |
| | 0 1 2 3 4 5 6 7 8 9 10<br>**Worst imaginable health while alive** ... **Best imaginable health** | |
| **D7.** | Please check the box that you believe most likely reflects your **cancer status ONE year** from now? | |
| | ☐1 | I will be cancer free (cured, remission, undetectable in the body) |
| | ☐2 | I will not be cancer free but my cancer will be controlled with treatment. |
| | ☐3 | My cancer will have spread but I am likely to be alive. |
| | ☐4 | I am unlikely to be alive. |
| **D8.** | We would like to know how good or bad your **health** will be in **ONE year**. This scale is numbered from 0 to 10. 10 means the best health you can imagine and 0 means the worst health you can imagine while still being alive. Indicate on the scale how your **health** will be in **ONE year**. | |
| | 0 1 2 3 4 5 6 7 8 9 10<br>**Worst imaginable health while alive** ... **Best imaginable health** | |
| **D9.** | Below is a list of statements that other people with your illness have said are important. Read each statement and please indicate one number per line to indicate the response which best describes how much you agree with that statement *right now*. | |

| | | **Strongly Disagree** | **Disagree** | **Agree** | **Strongly Agree** |
|---|---|---|---|---|---|
| 1. | I have a positive outlook towards life. | 1 | 2 | 3 | 4 |
| 2. | I have a short and/or long- range goals. | 1 | 2 | 3 | 4 |
| 3. | I feel alone. | 1 | 2 | 3 | 4 |
| 4. | I can see possibilities in the midst of difficulties. | 1 | 2 | 3 | 4 |

| | | | | | | |
|---|---|---|---|---|---|---|
| | 5. | I have faith that gives me comfort. | 1 | 2 | 3 | 4 |
| | 6. | I feel scared about my future. | 1 | 2 | 3 | 4 |
| | 7. | I can recall happy/joyful times. | 1 | 2 | 3 | 4 |
| | 8. | I have deep inner strength. | 1 | 2 | 3 | 4 |
| | 9. | I am able to give and receive caring/ love. | 1 | 2 | 3 | 4 |
| | 10. | I have a sense of direction | 1 | 2 | 3 | 4 |
| | 11. | I believe that each day has potential. | 1 | 2 | 3 | 4 |
| | 12. | I feel my life has value and worth. | 1 | 2 | 3 | 4 |

| SECTION E: PROGNOSIS COMMUNICATION | | |
|---|---|---|
| **E1.** | How important it is for you to know how your disease will affect your **quality of life**? | |
| | ☐1 | Very important |
| | ☐2 | Somewhat important |
| | ☐3 | A little important |
| | ☐4 | Not at all important |
| **E2** | How important it is for you to know how your disease will affect your **survival**? | |
| | ☐1 | Very important |
| | ☐2 | Somewhat important |
| | ☐3 | A little important |
| | ☐4 | Not at all important |
| **E3.** | How informed are you regarding how your **medical condition** is likely to **change over time**? | |
| | ☐1 | Very informed |
| | ☐2 | Somewhat informed |
| | ☐3 | Not informed |
| **E4.** | When did you last have a conversation with your doctor about how your health condition is likely to change over time? | |
| | ☐1 | More than 1 year ago |
| | ☐2 | 6 months to 1 year ago |
| | ☐3 | 3 to 6 months ago |
| | ☐4 | Less than 3 months ago |
| | ☐5 | Never |
| **E5**. | What has your doctor told you about the **primary** goal of your current treatment? | |
| | ☐1 | Cure my cancer |
| | ☐2 | Prolong my life |
| | ☐3 | Improve my symptoms |
| | ☐4 | Not sure |
| | ☐5 | Cannot recall |
| | ☐6 | He/She has not told me anything |
| | ☐7 | Others, please specify ___________________ |

| | | | | | |
|---|---|---|---|---|---|
| **E6.** | Have you ever discussed your expected survival with your doctor? | | | | |
| | ☐1 | Yes | | | |
| | ☐2 | No (Skip to Section F) | | | |
| **E7.** | **[If E6=1]** Does **your doctor** think you will be alive in …..? | | | | |
| | E7.1 | 20 years | | | |
| | | ☐1 | Yes (Skip to E8) | ☐2 | No |
| | E7.2 | 10 years | | | |
| | | ☐1 | Yes (Skip to E8) | ☐2 | No |
| | E7.3 | 5 years | | | |
| | | ☐1 | Yes (Skip to E8) | ☐2 | No |
| | E7.4 | 2 years | | | |
| | | ☐1 | Yes (Skip to E8) | ☐2 | No |
| | E7.5 | 1 year | | | |
| | | ☐1 | Yes (Skip to E8) | ☐2 | No |
| | E7.6 | 9 months | | | |
| | | ☐1 | Yes (Skip to E8) | ☐2 | No |
| | E7.7 | 6 months | | | |
| | | ☐1 | Yes | ☐2 | No |
| **E8.** | How did your doctor describe the survival outcomes? Check all that apply. | | | | |
| | ☐1 | In general terms (such as ‘a few months’ or ‘a few years’) | | | |
| | ☐2 | As likely length of survival (such as ‘patients with your condition live on an average 6 months’) | | | |
| | ☐3 | As chances of survival (‘10% over the next 5 years’) | | | |
| | ☐4 | In other terms, please specify________________ | | | |
| **E9.** | Has your doctor told you that even with the same treatment plan chances of survival vary for patients with similar health profiles? | | | | |
| | ☐1 | Yes | | | |
| | ☐2 | No | | | |

| SECTION F: PREFERENCES FOR PROGNOSTIC INFORMATION | | |
|---|---|---|
| **F1.** | Do you wish to obtain **further information** from your doctor about how your medical condition will **change overtime**? | |
| | ☐1 | Yes |
| | ☐2 | No |
| **F2.** | Do you wish to obtain **further information** from your doctor about how likely it is that your current treatment will **cure** your cancer? | |
| | ☐1 | Yes (Skip to F3) |
| | ☐2 | No |
| **F2.1** | **[If F2=2]** Why do you not wish to know from your doctor how likely it is that your current treatment will **cure your cancer**? Check all that apply. | |
| | ☐1 | The information will make me more distressed |
| | ☐2 | The information is unlikely to be accurate |
| | ☐3 | I am fearful of the emotional effects of the information on myself and my loved ones |
| | ☐4 | The information may make me lose hope |
| | ☐5 | I would want another person to receive the information |
| | ☐6 | I want to delay this conversation with my physician |
| | ☐7 | I have faith in my doctor's expertise |
| | ☐8 | I do not want to take too much of my doctor's time when other patients are waiting to be seen |
| | ☐9 | I do not want to focus on how long I will live |
| | ☐10 | I do not want to make this 'concrete.' |
| | ☐11 | My doctor does not know |
| | ☐12 | Others, please specify: _______________ |
| **F3.** | Do you wish to obtain **further information** from your doctor about **how long you are likely to live** under your current treatment? | |
| | ☐1 | Yes, in general terms (such as 'a few months' or 'a few years') (Skip to F4) |
| | ☐2 | Yes, in specific terms (such as 'on average 6 months' or 'chances of survival are 10% over the next 5 years') (Skip to F4) |
| | ☐3 | Yes, in other terms, please specify________________ (Skip to F4) |
| | ☐4 | No |
| **F3.1** | **[If F3=4]** Why do you not wish to know from your doctor **how long you are likely to live**? Check all that apply. | |
| | ☐1 | The information will make me more distressed |
| | ☐2 | The information is unlikely to be accurate |
| | ☐3 | I am fearful of the emotional effects of the information on myself and my loved ones |
| | ☐4 | The information may make me lose hope |
| | ☐5 | I would want another person to receive the information |
| | ☐6 | I want to delay this conversation with my physician |
| | ☐7 | I have faith in my doctor's expertise |
| | ☐8 | I do not want to take too much of my doctor's time when other patients are waiting to be seen |
| | ☐9 | I do not want to focus on how long I will live |
| | ☐10 | I do not want to make this 'concrete.' |
| | ☐11 | My doctor does not know |
| | ☐12 | Others, please specify: __________________ |

| | | |
|---|---|---|
| **F4.** | If your illness takes a turn for the worse and treatments are no longer beneficial, do you wish to be informed of the situation? | |
| | ☐1 | Yes |
| | ☐2 | No (Skip to F6) |
| **F5.** | **[If F4=1]** Who should be informing you of the situation if your illness takes a turn for the worse and treatments are no longer beneficial? Check all that apply. | |
| | ☐1 | Doctor or other healthcare professional |
| | ☐2 | Family members/ Friends |
| | ☐3 | Others, please specify: ______________________ |

| | | | | |
|---|---|---|---|---|
| **F6.** | Please indicate below whether you had sufficient information or you felt you needed more information when making the decision for your current treatment plan | | | |
| | | | **I felt I had sufficient information to help me decide which treatment I should take**<br><br>***Note:*** *If patient is not undergoing any active treatment, then ask:*<br>**I felt I had sufficient information to help me decide to forgo any active treatment** | **I felt I needed more information than what I had to help me decide which treatment I should take**<br><br>***Note:*** *If patient is not undergoing any active treatment, then ask:*<br>**I felt I needed more information than what I had to help me decide to forgo any active treatment** |
| | F6.1. | Information about how far advanced my cancer is | 1 | 2 |
| | F6.2. | Information about goals of my treatment | 1 | 2 |
| | F6.3. | Information about different treatment options for my cancer | 1 | 2 |
| | F6.4. | Information about advantages with each treatment option | 1 | 2 |
| | F6.5. | Information about disadvantages with each treatment option | 1 | 2 |
| | F6.6. | Information about cost for each treatment option | 1 | 2 |
| | F6.7. | Information about side-effects with each treatment option | 1 | 2 |
| | F6.8. | Information about uncertainty on whether or not the treatment will work | 1 | 2 |

| SECTION G: TREATMENT PREFERENCES | | |
|---|---|---|
| **G1.** | If you had to make a choice now, would you prefer treatment that extends life as much as possible, or would you want treatment that gives you minimal pain and discomfort? Please choose a point in the scale below. | |
| | 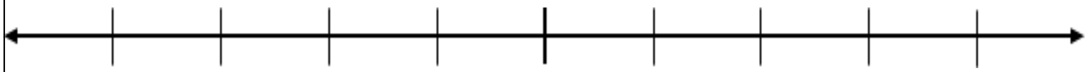 Extend life as much as possible; Severe pain or discomfort — Moderate life extension; Moderate pain or discomfort — No life extension; Minimal pain or discomfort | |
| **G2.** | If you had to make a choice now, would you prefer treatment that extends life as much as possible, or would you want treatment that costs you less? Please choose a point in the scale below. | |
| | 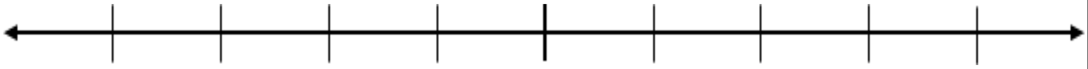 Extend life as much as possible; High cost — Moderate life extension; Moderate cost — No life extension; Less cost | |
| **G3.** | Have you discussed with your **family members** what treatment or care you would like to receive in the event you are no longer able to make decisions for yourself? | |
| | ☐1 | Yes |
| | ☐2 | No |
| **G4.** | Have you discussed with your **doctor** what treatment or care would you like to receive in the event you are no longer able to make decisions for yourself? | |
| | ☐1 | Yes |
| | ☐2 | No |
| **G5.** | If it became available, would you consider a new treatment strategy whose goal is to **reduce the 3 year MORTALITY rate by 5 percentage points**?<br><br>For instance, consider that with current/usual care being provided 90 out of 100 patients are likely to pass away in the next 3 years. This new treatment strategy will reduce mortality such that 85 out of 100 patients are likely to pass away in the next 3 years. | |
| | ☐1 | Very likely |
| | ☐2 | Likely |
| | ☐3 | Unlikely |
| | ☐4 | Very unlikely |

| SECTION H: ADDITIONAL BACKGROUND INFORMATION | | |
|---|---|---|
| **H1.** | Which ethnic group do you belong to? | |
| | ☐1 | Chinese |
| | ☐2 | Malay |
| | ☐3 | Indian |
| | ☐4 | Other, please specify ___________________________ |
| **H2.** | What is your religion? | |
| | ☐1 | Christian |
| | ☐2 | Buddhist |
| | ☐3 | Taoist |
| | ☐4 | Muslim |
| | ☐5 | Hindu |
| | ☐6 | Sikh |
| | ☐7 | Other, please specify ________________________________ |
| | ☐8 | No religion |
| | ☐9 | Free thinker |
| **H2.1** | **[If H2=1 to 7]** At a time of suffering and distress, to what extent do you find strength and comfort from your religion? | |
| | ☐1 | None |
| | ☐2 | A little |
| | ☐3 | Some |
| | ☐4 | A great deal |
| **H3.** | What is your current marital status? | |
| | ☐1 | Married |
| | ☐2 | Living with a partner |
| | ☐3 | Separated |
| | ☐4 | Widowed |
| | ☐5 | Divorced |
| | ☐6 | Never married |
| **H4.** | Do you have children? | |
| | ☐1 | Yes |
| | ☐2 | No |
| **H4.1** | **[If H4=1]** How many children do you have? ☐ | |
| **H5.** | Please indicate who (if any) you live with. You may check more than one box. | |
| | ☐1 | Self |
| | ☐2 | Spouse |
| | ☐3 | Children |
| | ☐4 | Parents |
| | ☐5 | Sibling |
| | ☐6 | Grandchildren |
| | ☐7 | Other, please specify __________________ |
| **H6.** | Do you have a primary caregiver who is most involved in providing you care (e.g. accompanies you for doctor's visits and helps you with day to day activities)? | |
| | ☐1 | Yes |
| | ☐2 | No |

| | | |
|---|---|---|
| **H6.1** | **[If H6=1]** What is your relationship with this person? This person is my: | |
| | ☐1 | Spouse |
| | ☐2 | Son |
| | ☐3 | Daughter |
| | ☐4 | Father |
| | ☐5 | Mother |
| | ☐6 | Brother |
| | ☐7 | Sister |
| | ☐8 | Grandparent |
| | ☐9 | Friend |
| | ☐10 | Other, please specify: ______________________ |
| **H7.** | Are you currently working? | |
| | ☐1 | Working full-time |
| | ☐2 | Working part-time |
| | ☐3 | Retired and not working |
| | ☐4 | Homemaker |
| **H8.** | Were you working before your illness? | |
| | ☐1 | Working full-time |
| | ☐2 | Working part-time |
| | ☐3 | Retired and not working |
| | ☐4 | Homemaker |
| **H9.** | What kind of health insurance coverage do you have? [Check all that apply] | |
| | ☐1 | None |
| | ☐2 | MediShield Life |
| | ☐3 | Integrated Shield Plan |
| | ☐4 | Private health insurance |
| | ☐5 | Others, please specify: ________________________ |
| **H10.** | How would you define your household's socio-economic status? | |
| | ☐1 | Poor |
| | ☐2 | Lower-middle income |
| | ☐3 | Middle income |
| | ☐4 | Upper-middle income |
| | ☐5 | High income |
| **H11.** | What is the total monthly household Income of your family from all sources (includes drawing down from savings)? | |
| | ☐1 | Less than $500 |
| | ☐2 | $500 - $999 |
| | ☐3 | $1,000 - $1,999 |
| | ☐4 | $2,000 - $2,999 |
| | ☐5 | $3,000 - $3,999 |
| | ☐6 | $4,000 - $4,999 |
| | ☐7 | $5,000 and above |
| | ☐8 | Don't know |
| **H12.** | What was the total monthly household income of your family from all sources (includes drawing down from savings) before the diagnosis of your illness? | |
| | ☐1 | Less than $500 |
| | ☐2 | $500 - $999 |
| | ☐3 | $1,000 - $1,999 |
| | ☐4 | $2,000 - $2,999 |
| | ☐5 | $3,000 - $3,999 |
| | ☐6 | $4,000 - $4,999 |
| | ☐7 | $5,000 and above |
| | ☐8 | Don't know |

| | | |
|---|---|---|
| **H13.** | What is your highest educational level completed? | |
| | ☐1 | No formal education |
| | ☐2 | Primary |
| | ☐3 | Secondary (including O Levels) |
| | ☐4 | Vocational/ITE |
| | ☐5 | Junior College/Polytechnic/Diploma |
| | ☐6 | University and above |
| | ☐7 | Don’t know/Can’t remember |

**Thank you for participating in the survey.**
